# Annual acknowledgement of reviewers

**DOI:** 10.1186/1471-2148-13-32

**Published:** 2013-03-07

**Authors:** Timothy R Sands

**Affiliations:** 1 BioMed Central, Floor 6, 236 Gray's Inn Road, London WC1X 8HB, UK

## Abstract

**Contributing reviewers:**

The editors of BMC Evolutionary Biology would like to thank all our reviewers who have contributed to the journal in Volume 12 (2012).

## 

Noritaka Adachi

Japan

Diane Adams

USA

Bayu Adjie

Japan

Gabriela Aguileta

Spain

Dirk Ahrens

UK

Diego Albani

Italy

Christian Albrecht

Germany

Miguel Alcaide

Canada

Benjamin Allen

USA

David Alvarez-Ponce

Spain

Andrei Alyokhin

USA

Ramón Anadón

Spain

Kirk Anderson

USA

Olin Anderson

USA

Jose Andres

Canada

Cecile Ané

USA

Nils Anthes

Germany

David R. Arahal

Spain

Roberto Arbore

Switzerland

Miguel Arenas

Spain

David Armisen

France

Wolfgang Arthofer

Austria

Raquel Assis

USA

Jan Axtner

Germany

Patricia Backwell

Australia

Justin Bahl

Singapore

Stuart Baird

France

William Baker

UK

Evgeniy Balakirev

USA

Francisco Balao

Spain

James Baldwin

USA

David Baltrus

USA

Pat Barclay

Canada

Tim Barraclough

UK

Malay Basu

USA

James Beck

USA

Andrew Beckerman

UK

Robert Belshaw

UK

Frida Ben-Ami

Israel

Edgar Benavides

USA

Philip Bergmann

USA

Daniel Berner

Switzerland

Coralie Bertheau

Austria

Giorgio Bertorelle

Italy

Bank Beszteri

Germany

Ricardo Betancur Rodriguez

Colombia

Mireille Betermier

France

Esther Betran

USA

Brian Bettencourt

USA

Rolf Georg Beutel

Germany

Etienne Bezault

USA

Martin Bidartondo

UK

Joseph Bielawski

Canada

Christian Biémont

France

Olaf Bininda-Emonds

Germany

Christopher Bird

USA

Pierre Bize

Switzerland

Mathieu Blanchette

Canada

Julien Bobe

France

Erich Bornberg-Bauer

Germany

Alex Bortvin

USA

Anke Braband

Germany

Dan Bradley

Ireland

Sean Brady

USA

Matthew Brandley

Australia

Douglas Brandon-Jones

Australia

Michael Braun

USA

Edward Braun

USA

Hans Breeuwer

Netherlands

Antonio Brehm

Portugal

Alberto Bressan

USA

Henner Brinkmann

Canada

Jennifer Brisson

USA

Daniela Brites

Switzerland

Luciano Brocchieri

USA

Michael Brockhurst

UK

Juergen Brosius

Germany

James Brown

UK

Ann Budd

USA

Alex Buerkle

USA

Marija Buljan

UK

Gordon Burleigh

USA

Thorsten Burmester

Germany

Ann Burnell

Ireland

Stephen Cairns

USA

Kevin Campbell

Canada

Ulrika Candolin

Finland

Aurore Canoville

South Africa

José Capitan

Spain

Margarida Cardoso Moreira

USA

Matt Carling

USA

Miguel Carneiro

Portugal

Miguel Carretero

Portugal

Vincent Castric

France

Matteo Cavaliere

Spain

Domitille Chalopin

France

Sylvain Charlat

France

Jane Charlesworth

UK

Deborah Charlesworth

UK

Bénédicte Charrier

France

Pierre-Olivier Cheptou

France

Michael Cherry

South Africa

Marcelo Cioffi

Brazil

Andrew Clark

USA

Marcus Clauss

Switzerland

Kendall Clements

New Zealand

David Close

Canada

Samuel Cobb

UK

Lachlan Coin

UK

Michael Collyer

USA

David Comas

Spain

Iñaki Comas

Spain

Jeffrey Conner

USA

Elena Conti

Switzerland

Natalie Cooper

Ireland

Tim Cooper

USA

Alan Cooper

Australia

Otto Xavier Cordero

Netherlands

Adrienne Correa

USA

Keith Crandall

USA

Andrew Crawford

USA

Teresa Crease

Canada

Chris Creevey

Ireland

Richard Culleton

Japan

Rute da Fonseca

Portugal

Tal Dagan

Germany

Domenico D'Alelio

Italy

Manuela d'Amen

Italy

Étienne Danchin

France

Savel Daniels

South Africa

Sharmishtha Dattagupta

Germany

William Davidson

Canada

Peter Davies

Canada

Steve Davis

USA

Vivian de Buffrénil

France

Arjan de Visser

Netherlands

Melanie Debiais-Thibaud

France

Ellen Decaestecker

Belgium

Charles Deeming

UK

Jacquelin DeFaveri

Finland

Frederic Delsuc

France

Erick Denamur

France

John Dennehy

USA

Miroslava Derenko

Russia

David Des Marais

USA

Tobias Deschner

Germany

Christophe Dessimoz

Switzerland

Giovanni Destro Bisol

Italy

Agnes Dettai

France

Jeremy Dettman

Canada

Sylvia Dewilde

Belgium

Xavier Didelot

UK

Thomas Diekwisch

USA

George Dimopoulos

USA

Anne-Ly Do

Germany

Kimberly Dodge

USA

Martin Dohrmann

USA

Russell Doolittle

USA

Marcel Dorken

Canada

Alex Dornburg

USA

Ivan Dotu

USA

Angela Douglas

UK

Nicole Dubilier

Germany

Siobain Duffy

USA

Meghan Duffy

USA

Julie Dunning Hotopp

USA

Micah Dunthorn

Germany

Lise Dupont

France

Melvin Duvall

USA

Paul Eady

UK

Ingo Ebersberger

Austria

Christopher Eckert

Canada

Greg Edgecombe

UK

Scott Edwards

USA

Scott Egan

USA

Thomas Eikbush

USA

John Eimes

USA

Christophe Eizaguirre

Germany

Robert Ekblom

Sweden

Omar Eldakar

USA

Bjarki Eldon

UK

Marianne Elias

France

Hans Ellegren

Sweden

Eyal Elyashiv

Israel

Laura Eme

Canada

Richard Emes

UK

Giovanni Emiliani

Italy

Johannes Engelken

Spain

Jan Engelstaedter

Australia

Leif Engqvist

Germany

David Erickson

USA

Bente Eriksen

Sweden

Jose Antonio Escudero

France

Ben Evans

Canada

Pierre-Henri Fabre

Denmark

Andrzej Falniowski

Poland

Daniel Falush

UK

Ashley Farlow

Austria

Heike Feldhaar

Germany

Joe Felsenstein

USA

David Ferrier

UK

David Fewer

Finland

Reiner Finkeldey

Germany

Anna Maria Fiore-Donno

Germany

Mark Fishbein

USA

Kate Fitzgerald

USA

Courtney Fitzpatrick

USA

Martin Flajnik

USA

Ian Fleming

Canada

Jean-François Flot

Germany

Jessica Fong

USA

Diego Fontaneto

Italy

Felix Forest

UK

Kristoffer Forslund

Sweden

Zac Forsman

USA

Antoine Fouquet

Brazil

Scott France

USA

Ceridwen Fraser

Belgium

Ruth Freire

Spain

Joachim Frommen

Switzerland

Jérôme Fuchs

France

Jesualdo Fuentes

USA

Toni Gabaldon

Spain

Jürgen Gadau

USA

Juan Galindo

Spain

Miguel Gallach

Austria

Sonia Garcia

Spain

Santi Garcia-Vallvé

Spain

Andy Gardner

UK

John Gatesy

USA

Eric Gaucher

USA

Marcelo Gehara

Germany

Jonathan Geisler

USA

Martin Genner

UK

Justin Gerlach

UK

Lena Gerwick

USA

Michael Ghedotti

USA

Godelieve Gheysen

Belgium

Mary Gibby

UK

Katherine Gibson

USA

Alfons Gierl

Germany

Dimitri Gilis

Belgium

Tatiana Giraud

France

Gonzalo Giribet

USA

Marc Girondot

France

Chris Gissendanner

USA

Carmela Gissi

Italy

Gernot Glöckner

Germany

Greg Gloor

Canada

J Peter Gogarten

USA

Hemalatha Golaconda Ramulu

France

Debra Goldberg

USA

Brian Golding

Canada

Africa Gomez

UK

Jesus Gomez-Zurita

Spain

Fernando Gonzalez-Candelas

Spain

Alexander Gorbalenya

Netherlands

Isabel Gordo

Portugal

Swanne Gordon

Finland

Jeff Gore

USA

Sven Gould

Germany

Mikhail Grachev

Russia

Barbara Gravendeel

Netherlands

Beverley Green

Canada

Alexander Gruhl

UK

Ivana Gudelj

UK

Yan-Ping Guo

China

Yalong Guo

China

Darryl Gwynne

Canada

Christoph Haag

Switzerland

Alex Hall

Switzerland

Barry Hall

USA

Benedikt Hallgrimsson

Canada

Leng Han

USA

Jing Han

China

L. Curtis Hannah

USA

Anne Harduin-Lepers

France

Gordon Harkins

South Africa

Luke Harmon

USA

Adrian Harwood

UK

Steffen Harzsch

Germany

Graham Hatfull

USA

Mark Hauber

USA

Bernhard Hausdorf

Germany

David Haymer

USA

Chris Heesy

USA

Hanna Heidel-Fischer

Germany

George Heimpel

USA

Jochen Heinrichs

Germany

Fabrice Helfenstein

Switzerland

Ines Hellmann

Austria

Jakob Hemmer-Hansen

Denmark

Jan Michael Hemmi

Australia

Frederico Henning

Germany

Russell Hermansen

USA

Katja Heubel

Germany

Megan Higgie

Australia

Geoffrey Hill

USA

Khidir Hilu

USA

Kerstin Hoef-Emden

Germany

Bert Hoeksema

Netherlands

Jason Hoeksema

USA

Ary Hoffmann

Australia

Michael Hofreiter

Germany

Paul Hohenlohe

USA

Linda Holland

USA

Brenden Holland

USA

Johan Hollander

Sweden

Michael Hood

USA

Iñaki Hormaza

Spain

Michael Horn

USA

Emily Hornett

USA

Veit Hornung

Germany

David Houle

USA

Richard Howard

USA

Howard Howland

USA

Huateng Huang

USA

Jinling Huang

USA

Alan Hudson

UK

Andrew Hugall

Australia

John Hunt

UK

Arild Husby

Norway

Katriina Ilves

USA

Vivian Irish

USA

David Irwin

Canada

Lakshminarayan Iyer

USA

Jef Jaeger

USA

Axel Janke

Germany

Daniel Jeffares

UK

Chris Jiggins

UK

Frank Johansson

Sweden

Norman Johnson

USA

Michele Johnson

USA

Jill Johnson

USA

Marc Jolly

UK

Emily Jones

USA

Luca Jovine

Sweden

Carlos Juan

Spain

Andrey Kajava

France

Ilias Kappas

Greece

Elinor Karlsson

USA

Robert Karn

USA

Einat Karpestam

Sweden

Masahiro Kato

Japan

Sigrid Katz

USA

Akito Yuji Kawahara

USA

Shinpei Kawaoka

Japan

Jukka Kekäläinen

Finland

Thomas Keller

USA

Gael Kergoat

France

Sander Kersten

Netherlands

Valerio Ketmaier

Germany

Hossein Khiabanian

USA

Taisei Kikuchi

Japan

C. William Kilpatrick

USA

Eunsuk Kim

USA

Kayla King

UK

Evan Kingsley

USA

Michael Kinnison

USA

Karin Kiontke

USA

Roland Kirschner

Taiwan

Hirohisa Kishino

Japan

Ralf Kittler

USA

Karl Kjer

USA

Steffen Klaere

New Zealand

Leszek Kleczkowski

Sweden

Hope Klug

USA

Annette Klussmann-Kolb

Germany

Rob Knell

UK

Stephan Koblmüller

Austria

Joshua Kohn

USA

Masahiro Kono

USA

Ryszard Korona

Poland

Carolin Kosiol

Austria

Britt Koskella

UK

Marta Kostrouchova

Czech Republic

Ales Kovarik

Czech Republic

Zbynek Kozmik

Czech Republic

Indrikis Krams

Latvia

Matthew Krasowski

USA

Brian Kreiser

USA

Daniel Kronauer

USA

Shawn Kuchta

USA

Rob Kulathinal

USA

Justin Kumar

USA

Krushnamegh Kunte

India

Alexander Kupfer

Germany

Atsushi Kurabayashi

Japan

Shigehiro Kuraku

Japan

Elizabeth Kutter

USA

Minna-Maarit Kytöviita

Finland

Maurizio Labbate

Australia

Thurston Lacalli

Canada

Jason Ladner

USA

Daniel Lahr

Brazil

Simon Lailvaux

USA

Anna-Liisa Laine

Finland

Mariusz Lamentowicz

Poland

Kathrin Lampert

Germany

B. Franz Lang

Canada

Michael Lang

France

Hayley Lanier

USA

Dan Larhammar

Sweden

Peter Larsen

USA

Karl-Henrik Larsson

Norway

Amparo Latorre

Spain

Matt Lavin

USA

Daniel Lawson

UK

Julie Lee-Yaw

Canada

Niles Lehman

USA

Andrew Leitch

UK

Frederik Leliaert

Belgium

Bernardo Lemos

USA

Tobias Lenz

USA

Richard Leschen

New Zealand

Delano Lewis

USA

Li Liao

USA

Kai Lindström

Finland

Terje Lislevand

Norway

Tao Liu

China

Hongmei Liu

China

Jianquan Liu

China

Leila Lo Leggio

Denmark

Ben Longdon

UK

Andres Lopez

USA

Xavier Lopez

Spain

Francisco López de Saro

Spain

Stephen Lougheed

Canada

Francisco Lozano

Spain

Jian Lu

USA

Stefan Luepold

USA

Oksana Lukjancenko

Denmark

Magnus Lundberg

Hong Kong

Nina Lundholm

Denmark

Francois Lutzoni

USA

Petros Lymberakis

Greece

Jeremy Lynch

USA

Denis Lynn

Canada

Sinéad Lyons

UK

Martine Maan

Netherlands

Kohji Mabuchi

Japan

Milos Macholan

Czech Republic

Constantino Macias Garcia

Mexico

Dan Macqueen

UK

Christine Maggs

UK

Karl Magnacca

USA

Kira Makarova

USA

Takashi Makino

Japan

Ludovic Mallet

France

Diego Mallo

Spain

Ferruccio Maltagliati

Italy

Anup Mandal

India

Stéphanie Manel

France

Paul Manger

South Africa

Mollie Manier

USA

Paul Manos

USA

Ralph Marcucio

USA

Paul Marek

USA

John Margaritopoulos

Greece

David Mark Welch

USA

Joseph Marsh

UK

Jeremy Marshall

USA

Luis Alberto Martinez Vaquero

Spain

Andrés Martínez-Aquino

Mexico

Alino Martinez-Marcos

Spain

Ryuichi Masuda

Japan

Julieta Mateos

Germany

Mariana Mateos

USA

Sarah Mathews

USA

Conrad Matthee

South Africa

Daniel Matute

USA

Laura May-Collado

USA

John McCormack

USA

Alan McNally

UK

Patrick Meirmans

Netherlands

Richard Meisel

USA

Joseph R Mendelson III

USA

Joachim Mergeay

Belgium

Natacha Mesquita

Portugal

Rachel Meyer

USA

Claire-Lise Meyer

Belgium

Yvonne Meyer-Lucht

Sweden

Jun Miao

USA

Andy Michel

USA

Borja Mila

Spain

Michel Milinkovitch

Switzerland

Robert Miller

USA

David Miller

Australia

Geoffrey Miller

USA

Suzanne Mills

France

Aurélien Miralles

Germany

Dan Mishmar

Israel

Carolina Mizuno

Spain

Michael Möller

UK

Stefano Mona

France

Philippe Monget

France

Valeria Montano

Austria

Fernando Montealegre-Zapata

UK

Marina Montresor

Italy

Richard Moore

USA

Patricia Moore

USA

Andrew Moore

Germany

Jiri Moravec

Czech Republic

Yolanda Morbey

Burkina Faso

David Moreira

France

Mary Morgan-Richards

New Zealand

Levi Morran

USA

Molly Morris

USA

Rachel Mueller

USA

John Mulley

UK

José Mullor

Spain

Jeong-Hwan Mun

South Korea

Robert Murphy

Canada

Moritz Muschick

Switzerland

Arcady Mushegian

USA

Jongmin Nam

USA

Francesco Nardi

Italy

David Navarro

Spain

Jesus Navas-Castillo

Spain

Volker Nehring

Germany

Maurine Neiman

USA

Andre Nel

France

David Nelson

USA

Camilla Nesbo

Canada

Alejandro Nettel

USA

Kurt Neubig

USA

Matthew Niemiller

USA

Nikolas Nikolaidis

USA

Arne Nolte

Germany

Ryan Norris

USA

Jamie Oaks

USA

Darren Obbard

UK

Karen Ober

USA

Dietrich Ober

Germany

Miroslav Obornik

Czech Republic

Mary O'Connell

Ireland

Brian Oliver

USA

Richard Oliver

Australia

Mats Olsson

Australia

Juan Opazo

Chile

Yaara Oren

Israel

Claudia Patricia Ornelas-Garcia

Mexico

Pablo Orozco ter Wengel

UK

Csaba Ortutay

Finland

Benjamin Oswald

USA

Martin Paeckert

Germany

Anna Panchenko

USA

John Pannell

Switzerland

Jordi Paps

UK

Dimitrios Paraskevis

Greece

Aristeidis Parmakelis

Greece

John Parsch

Germany

Yale Passamaneck

USA

Marco Passamonti

Italy

Michael Patten

USA

Octavio Paulo

Portugal

Ovidiu Paun

Austria

Maxime Pauwels

France

Teresa Pawlowska

USA

Carlos Peña

Finland

Rafael Penafiel

Spain

Junhua Peng

China

Bridget Penman

UK

David Penny

New Zealand

Javier Perez-Tris

Spain

Susan Perkins

USA

Gabriel Perron

USA

Bengt Persson

Sweden

A. Townsend Peterson

USA

Markus Pfenninger

Germany

Tim Phillips

UK

João Pinto

Portugal

Helen Piontkivska

USA

Paolo Piras

Italy

Andre Pires-daSilva

USA

Ricardo Pita

Portugal

Gordon Plague

USA

Helmut Plattner

Germany

Federico Plazzi

Italy

Lars Podsiadlowski

Germany

Grant Pogson

USA

Hendrik Poinar

Canada

Julia Poncela-Casasnovas

USA

Ovidiu Popa

Germany

Susannah Porter

USA

Erik Postma

Australia

Daniel Potter

USA

Rodolphe Poupardin

UK

Jeff Powell

Australia

Angelika Preisfeld

Germany

Elizabeth Prendini

USA

Tom Price

UK

Trevor Price

USA

Dana Price

USA

Nicholas Priest

UK

Christian Printzen

Germany

Tal Pupko

Israel

Pengfei Qi

China

Hong Qin

USA

Jorge Quarleri

Argentina

Petr Ráb

Czech Republic

Nishanta Rajakaruna

USA

Steven Ramm

Germany

Sebastian Ramos-Onsins

Spain

David Rand

USA

Bruce Rannala

USA

Vincent Ranwez

France

Olga Raskina

Israel

Christopher Raxworthy

USA

James Raymond

USA

Sylvain Razafimandimbison

Sweden

Rodrigo Redondo

Austria

Sebastien Renaut

Canada

Sylvie Retaux

France

Liam Revell

USA

Ignacio Ribera

Spain

David Richardson

UK

Stefan Richter

Germany

Cynthia Riginos

Australia

Trevor Rivers

USA

Dale Roberts

Australia

Hugh Robertson

USA

Phyllis Robinson

USA

James Rodger

South Africa

Buel Rodgers

USA

Francisco Rodriguez-Valera

Spain

Marius Roesti

Switzerland

D. Christopher Rogers

USA

Sean Rogers

Canada

Adam Rollins

USA

Jose Romero

Spain

Marianne Rooman

Belgium

Cristina Roquet

France

Mary Rorick

USA

Laura Rose

Germany

Gil Rosenthal

USA

Omar Rota-Stabelli

Italy

Olivia Roth

Germany

Carl Rothfels

USA

Julien Roux

USA

Scott Roy

USA

Nick Royle

UK

Daniel Rozen

UK

Nimrod Rubinstein

Israel

Manuel Ruedi

Switzerland

Alfredo Ruiz

Spain

Suzanne Rutherford

USA

Daniel Ruzzante

Canada

Kalle Rytkonen

USA

Niv Sabath

Israel

Timothy Sackton

USA

Nicolas Salamin

Switzerland

Angel Sánchez

Spain

Alicia Sanchez-Mazas

Switzerland

David Sandeman

Germany

Ian Sanders

Switzerland

Rafael Sanjuán

Spain

Isabel Sanmartin

Spain

Juan Santos

USA

Francisco Santos

Portugal

Tiina Särkinen

UK

Pierre Sasal

France

Simon Saule

France

Richard Saunders

Hong Kong

Massimo Scandura

Italy

Lukas Schärer

Switzerland

Enrico Schleiff

Germany

Amy Schmid

USA

Andreas Schmidt-Rhaesa

Germany

Juergen Schmitz

Germany

Gerald M. Schneeweiss

Austria

Harald Schneider

UK

Jan Schnitzler

Germany

Torsten Schöneberg

Germany

Sijmen Schoustra

Netherlands

Trina Schroer

USA

Hinrich Schulenburg

Germany

Andreas Schüler

Germany

Katharina Schulte

Australia

Ted Schultz

USA

Giovanni Scopece

Italy

John Scott

USA

Charlie Scutt

France

Satoshi Sekimoto

USA

Noa Sela

Israel

Juan Carlos Senar

Spain

Jeanne Serb

USA

Ester Serrao

Portugal

Hongyan Shan

China

Itai Sharon

USA

Mark Shaw

UK

A. Jonathan Shaw

USA

Nancy Sherwood

Canada

Suhua Shi

China

Sebastian Shimeld

UK

Takahiro Shiotsuki

Japan

Shin-Han Shiu

USA

Joel Shore

Canada

Jeffrey Shultz

USA

Olin Silander

Switzerland

James Simmer

USA

Skuli Skulason

Iceland

Jon Slate

UK

Montgomery Slatkin

USA

Michel Slotman

USA

Carole Smadja

France

Per Smiseth

UK

Joe Smith

USA

Paul Smith

USA

David Roy Smith

Canada

Terry Snell

USA

Niko Sonnenschein

USA

Michael Sorenson

USA

Ulf Sorhannus

USA

Teiji Sota

Japan

Felix Sperling

Canada

Stevan Springer

USA

Mark Springer

USA

Ted Stankowich

USA

Michael Stat

Australia

Spencer Staton

USA

Kelly Steele

USA

Diana Stein

USA

Sebastian Steinfartz

Germany

Steve Stephenson

USA

Frank Stewart

USA

Michael Stich

Spain

David Stock

USA

Jaroslaw Stolarski

Poland

Anne Stone

USA

Tanja Strand

Sweden

Jared Strasburg

USA

Axel Strauß

Germany

Jeffrey Streicher

USA

Romain Studer

UK

David Studholme

UK

Eva Stukenbrock

Germany

Zhixi Su

China

Kenta Sumiyama

Japan

Michael Sundue

USA

Thomas Owens Svennungsen

Norway

Graeme Swindles

UK

Kazuhiro Takemoto

Japan

Corina Tarnita

USA

Oznur Tastan

Turkey

Martin Taylor

UK

Sabrina Taylor

USA

Aurélien Tellier

Germany

John Terblanche

South Africa

Olaf Thalmann

Finland

Jeffrey Thorne

USA

Elisabeth Tillier

Canada

Nelson Ting

USA

Joseph Tobias

UK

David Toews

Canada

Sergio Tofanelli

Italy

Hirokazu Toju

Japan

Masayoshi Tokita

USA

Charlotte Tollenaere

Finland

Ralph Tollrian

Germany

Clara Torres-Barceló

UK

Stefano Torriani

Switzerland

Vladimir Trifonov

USA

Hirokazu Tsukaya

Japan

Pablo Tubaro

Argentina

Tobias Uller

UK

Sylvain Ursenbacher

Switzerland

Vladimir Uversky

USA

Victor D. Vacquier

USA

Pedro Vale

France

Luis Valente

UK

Alain Vanderpoorten

Belgium

Sergio Vargas

Costa Rica

Chris Venditti

UK

George Verboom

South Africa

Dhanasekaran Vijaykrishna

Singapore

Jakob Vinther

USA

Claudia Voelckel

New Zealand

Tom Vogwill

UK

Pascal Vonlanthen

Switzerland

Elena Voronezhskaya

Russia

Jeromos Vukov

Portugal

Michael Wade

USA

Andrea Waeschenbach

UK

Philipp Wagner

USA

Giselle Walker

New Zealand

Stefan Walker

Australia

Ross Frederick Waller

Australia

Graham Wallis

New Zealand

Qiu-Hong Wan

China

Zhen Wang

China

Wen Wang

China

Ian Wang

USA

Ing-Nang Wang

USA

Daryi Wang

Taiwan

Glenda Wardle

Australia

John Wares

USA

Yasuyuki Watano

Japan

Karen Wawrousek

USA

Stefan Weckx

Belgium

Michael Weiß

Germany

Jun Wen

USA

Joel Wertheim

USA

Helena Westerdahl

Sweden

Erica Westerman

USA

Christopher Wheat

Finland

Tom White

USA

James Whitfield

USA

Danielle Whittaker

USA

Brian Wiegmann

USA

Shawn Wilder

Australia

Gerald Wilkinson

USA

Lance Williams

USA

Tom Williams

UK

Matthew Albion Wills

UK

Luzie Wingen

UK

Stephen Withers

Canada

Jason Wolf

UK

Arielle Woznica

USA

Qingfa Wu

China

Bin Wu

China

Haibing Xie

China

Guangming Xiong

Germany

Zujun Yang

China

Shigeki Yasumasu

Japan

Sam Yeaman

Canada

Soojin Yi

USA

Jun Yu

China

Bisong Yue

China

Frank Zachos

Austria

Absalom Zamorano

Mexico

Yuan-Ming Zhang

China

Yong Zhang

USA

Wenheng Zhang

USA

Hongyu Zhang

China

Ziming Zhao

USA

Olga Zhaxybayeva

USA

Ying Zhen

USA

Robert Zink

USA

Christian Zmasek

USA

Roman Zug

Germany

Manuel Zúñiga

Spain

